# Vitamin D serum level in participants with positive history of recurrent herpes labialis

**DOI:** 10.1186/s12903-023-02924-0

**Published:** 2023-04-20

**Authors:** Zahra Ranjbar, Fatemeh Lavaee, Maryam Karandish, Farnaz Peiravian, Fateme Zarei

**Affiliations:** 1 Oral and Dental Disease Research center, Oral and Maxillofacial Disease Department, School of Dentistry, Shiraz University of Medical Sciences, Shiraz, Iran; 2 Department of Orthodontics, School of Dentistry, Shiraz University of Medical Sciences, Shiraz, Iran; 3 Student research committee, Shiraz University of Medical Sciences, Shiraz, Iran

**Keywords:** Recurrent herpes labialis, Vitamin D, Serum

## Abstract

**Aim:**

Vitamin D plays an important role in immune system regulation, also its deficiency is assumed to affect the patients’ predisposition to viral diseases such as recurrent herpes labialis. In this cross-sectional study, we tried to compare the mean serum level of vitamin D in participants with a positive history of recurrent herpes labial lesions and healthy controls.

**Materials and methods:**

The vitamin D serum level of 43 participants with a positive history of recurrent herpes labial lesions who were referred to the Motahhari laboratory in Shiraz during 2020–2022, was compared with 42 healthy controls. It was assessed by an Elisa kit. An Independent T-test was used to compare the vitamin D serum level between two genders. In order to assess the mean age value and gender distribution, an independent T-test and Pearson Chi-Square were used, respectively for the two groups. The serum vitamin D level was compared between both control and test groups.

**Results:**

There was no significant difference between vitamin D mean serum levels in the two evaluated groups (p.value = 0.72). Although the age (p.value = 0.09) and recurrence (p.value = 0.13) of herpes labialis had no statistically significant relation to the vitamin D serum level, the healing duration of herpes labialis was inversely related (p.value = 0.01). Lower-level of serum vitamin D were accompanied by a longer healing duration of the lesions.

**Conclusion:**

Although the vitamin D serum level of participants with a history of recurrent herpes labialis had no relation with age and herpes virus recurrence frequency, a longer healing duration of lesions had been reported in patients with lower serum levels of vitamin D.

## Introduction

Recurrent Herpes Labialis (RHL) is one of the most common oral vesiculoulcerative viral lesions [1]. The most common herpetic oral lesions are classified into two types HSV-1(Herpes simplex virus) [2] and HSV-2. Basically, HSV-1 usually involves above the waist while HSV2 is responsible for genital infections. Recurrent HSV infections can be stimulated by stress, upper respiratory tract infection, fever, ultraviolet light, trauma, and immune incompetence [3–6].

Vitamin D as a fat-soluble secosteroid, is a dietary supplement and naturally presents in a few foods. promotes calcium, magnesium, and phosphate intestinal absorption. Active metabolites are produced whether the sun rays strike the skin resulting in the completion of the photosynthetic process of vitamin D3 or consuming foods rich in Vitamins D2 and vitamin D3 hydroxylated by the cytochrome P450 enzyme in liver and CYP27B1 enzyme in the kidney to construct 1,25(OH)2D or calcitriol. This is the final active product in the human body [2].

Collectively, the innate immune system is reinforced and the adaptive immune system is controlled by 1,25(OH)2D [7, 8]. In general, Vitamin D sufficiency plays a critical role having a practical immune response in endocrine system. In addition, 1,25-dihydroxyVit amin D3 (1,25(OH)2D3), has also some effect on the modulation of immune system. T helper type 1 (Th1) is essential in immune responses in order to attack and the pathogens. Th1 remove the pathogens by increasing interferon (IFN)-γ and reducing interleukin (IL-4) [9].

Some vitamin D disturbances are reported in literature for patients who had been affected by viral diseases. Amongst viral infections, Sali et al. reported the lower serum level of vitamin D in patients with chronic hepatitis B [9]. Emanuel Zitt et al. in other study concluded that in a poor antibody formation against hepatitis B immunization, vitamin D deficiency was detected [10].

Due to scarce studies on the serum level of vitamin D in patients with recurrent herpes simplex, and controversial findings about the relation of vitamin D serum level and incidence of viral diseases, in this study we evaluated vitamin D serum level in patients with recurrent herpes labialis.

## Method and material

This cross-sectional study, during 2020–2021, was conducted according to the ethical principles of Helsinki [11] and was approved by the Ethics committee of Shiraz University of Medical Sciences (Ethics Code: IR.SUMS.DENTAL.REC.1398.076). The participants more than 18 years old were categorized into two groups.

43 Patients with the history of at least 3 times recurrence of herpes labialis (RHL) during the past year who had been referred to Shiraz Dental Faculty with an expert specialist confirmation had been enrolled in the case group. The excluding criteria were the history of systemic and immunological diseases that might affect the immune system and tissue healing such as diabetes mellitus, taking dietary supplements and pregnancy.

Another 42 healthy subjects with no history of any recurrent herpes simplex lesion had been entered in the control group.

All the participants signed the written informed consent form. Demographic data, gender, age, history of serious illness, any medication, the recurrence rate of RHL and its healing duration were registered for all participants. The healing period of lesions are mentioned from the time of.

### RHL appearance until lesion disappearance

Venous blood samples (5 mL) were taken in a time in which the participants didn’t have any active RHL, after a period of fasting at night by an expert nurse. The samples were delivered to laboratory.

Samples were allowed to clot and sera were separated by centrifugation (3500 rpm, 20 min, 25° C) then they had been stored at 20° C until use for analysis with the Vitamin D Elisa kit (PADTAN GOSTAR ISAR CO.). The incidence of RHL and its frequency in patients and their relation to the serum level of vitamin D was assessed. The serum level of vitamin D was evaluated considering the incidence of RHL and its frequency.

The data was analyzed by SPSS version 18. Pearson Chi-Square test was used for assessing the gender distribution of both groups. Student T test was used for comparing the mean age and vitamin D serum level of participants in both groups. Correlation test was used in order to assess the frequency and healing period.

## Results

In this cross-sectional study, 43 patients with history of recurrent herpes labialis 33 women and 10 men were enrolled. In control group 42 healthy participants without any history of recurrent herpes labialis participated. There was no significant difference between sex distribution of both groups’ participants(p.value = 0.41) (Table 1).

The mean age of patients with positive herpes labialis history and in healthy control group were 37.28 ± 9.19 and 30.86 ± 12.19 years old respectively. The age range of participants in 2 evaluated groups were statistically different (p.value = 0.007) (Table 1).

**Table 1:** The demographic data of both groups

Group	Sex		Total	P.value	Age (years old) mean ± SD	P p.value
	Men	Women				
RHL	10 23.3%	33 76.7%	43	0.41	37.28 ± 9.19	0.007
Control	8 19%	34 81%	42		36.17 ± 12.23	

The mean serum level of vitamin D in both groups are reported in Table 2.

**Table 2:** the mean serum level of vitamin D in both groups

Groups	Number	Mean(ng/ml)	Std.Deviation	P.value
Patients with history of RHL	43	24.27	12.57	0.72
Healthy control	42	23.21	14.79	

There was no significant difference between vitamin D mean serum level in the two evaluated groups (p.value = 0.72).

**Table 3:** The relation of healing period and recurrence of RHL with serum vitamin D serum level

Group	Min (Day)	Max(day)	Mean ± SD day	Pearson correlation	P.value
Healing period	7.5	15.5	10.05 ± 2.13	0.132	0.13
Recurrence of RHL	3 times/year	10 times/year	5.26 ± 2.51	− 0.013	0.01

Although the age (p.value = 0.09) and recurrence (p.value = 0.13) of herpes labialis had no statistically significant relation to the vitamin D serum level, the healing duration of herpes labialis was inversely related (p.value = 0.01) (Table 3). Lower-level of serum vitamin D was accompanied by longer healing duration of the lesions.

## Discussion

Vitamin D serum level was not different between both evaluate groups. Based on the results of this study, the mean serum level of vitamin D was not statistically different between the patients with history of RHL and the control groups without history of RHL. Although there was no relation between age and recurrence frequency of RHL and vitamin D serum level, the longer healing duration was accompanied by lower vitamin D serum level.

The relation of healing duration of herpetic lesions and serum level might be related to the complementary effect of vitamin D on immunologic behavior of herpes virus.

To the best of our knowledge, HSV infection inhibits Tumor necrosis factor- α (TNF-α) as a vital cytokine in the innate immunity. This cytokine induces expression of genes involved in inflammatory response [12]. On the other hand, histopathological evaluations in an animal model confirmed that vitamin D deficiency can cause delay in tissue healing [13]. Vitamin D can affect tissue healing by immune regulation, inflammation reduction and oxidative stress [14]. Active vitamin D stimulates macrophage phagocytosis and bacterial killing [15] and is a potent suppressor of the interferon-γ secretion by macrophages [16]. Vitamin D also induces T cell proliferation suppression and T helper type 1 cytokines reduction and increases production of T helper type 2 cytokines [17], which can improve wound healing.

According to the previous studies, there is no study about the relation of serum level and RHL. There are some evaluations about other viral diseases and vitamin D serum level which are controversial.

The antimicrobial effect of vitamin D supplementation has been explained in previous studies for viral disease such as respiratory infections, HIV and hepatitis B [18]. Some studies reported a lower level of in patients with hepatitis B. They also proposed that high level of viral load in these patients was related to vitamin D deficiency [19]; However, Jones G. et al. did not show any relation [20].

A study proposed that low serum level of vitamin D in patients with hepatitis B might induce higher replication level of hepatitis B virus [21]. In a study INF-α treatment for hepatitis B combined with vitamin D supplementation were more effective than INF-α alone in an animal model [22].

In another study, vitamin D deficiency has been suggested to be accompanied with weak immunity reactions such as seroconversion and protection for hepatitis B vaccination [10]. In contrast, another study reject the immunity enhancement by calcitriol injection after influenza vaccination [23].

Goncalves-Mendes et al. in a study evaluated the effect of receiving cholecalciferol before influenza vaccination [24].They reported higher level of TGF-β and lower level serum of TNF-α, IL-6 after influenza vaccination without antibody production improvement [24]. The results were confirmed by two other studies [23, 25].

Greiller et al. proposed that vitamin D metabolites cannot affect the rhinovirus, respiratory syncytial virus and influenza virus replication or clearance [26]. In contrast Zdrenghea et al. suggested vitamin D as a supplementation for acute respiratory infection prevention and treatment [27]. Another assessment suggested vitamin D supplementation for H1N1-induced influenza suppression [23]. This antiviral property was confirmed for EBV in another study [28].

Mandorfer et al. correlated the liver disease progression in HIV/HCV co-infected patients with low vitamin D level [29].

As the same as some of these studies which did not confirm the relation of and viral diseases, our findings were not different between healthy controls with no history of RHL and participants with history of RHL.

Vitamin D is a secosteriol which can act as a signaling pathway for immune system regulation [30]. Vitamin D receptors are commonly expressed on immune cells [31]. Monocytes, macrophages and dendritic cells can secrete 1-αhydroxylase an activator enzyme for 25(OH)D conversion to calcitriol [32]. Calcitriol prevent some pre-inflammatory mediators such as interferon- γ and tumor necrosis factor-α [14, 15, 33]. Vitamin D can shift the immune response from Th1 to Th2 cell response and it can decrease the inflammation by inducing immunosuppression [14, 16, 17].

Vitamin D deficiency impair macrophage maturation, expression of specific surface antigen and antimicrobial function [34, 35]. So vitamin D might have an important effect in immune response modulation of infectious disease [24].

There are some evidences in literature about the human microbiota and its potential in dication of diagnostic biomarkers which can be an indicator of normal or pathologic reactions, therapeutic response [36–38]; also the human microbiota can affect and shape the immune system [39].

Considering the immune regulatory and antimicrobial effects of vitamin D, the controversial comments about its role in treatment or prevention of viral disease is challenging.

Different viruses with different pathogenicity and virulence factors affect the immune responses through different pathway [40]. Also, each viral disease has different stages and previous studies have been performed in different stages.

Different kits which are used in laboratories with diverse range of normal level of vitamin D also might be deliberate as an effective factor.

Aging decreases the active form of vitamin D by 50%, this is due to decrease in renal function and calcium absorption, although secondary hyperparathyroidism is a compensatory mechanism for maintaining the serum vitamin D level Vitamin D and Aging [41].The discrepancy of age of participants in two evaluated groups is worthy to be considered. Although the age of participants with RHL was higher than healthy ones which presumed to have lower serum level of vitamin D, regarding their age, there was no difference between two evaluated groups. This can be related to the compensatory mechanism of body and the young age range of participants which are both in the same level approximately.

Cultural, nutritional factors for each community in addition to the individual immunity condition of each person can affect the results.

Assessing the vitamin D serum level in specific phase of viral infection for all participants makes the results more comparable. The participants of this study were not in the active phase of RHL at the time of blood sample preparation; but larger sample size can be suggested for future evaluations.

## Conclusion

Although vitamin D serum level of participants with history of recurrent herpes labialis had no relation with age and herpes virus recurrence frequency, longer healing duration of lesions had been reported in patients with lower serum level of vitamin D.

## Data Availability

The datasets used and/or analyzed during the study are available from the corresponding author on reasonable request.

## List of abbreviations

RHL: Recurrent Herpes Labialis
HSV: Herpes simplex virus
TNF-α: Tumor necrosis factor-α
Th1: T helper type 1
IFN: Interferon
